# LncRNA Functional Screening in Organismal Development

**DOI:** 10.3390/ncrna9040036

**Published:** 2023-06-28

**Authors:** Yang Li, Huicong Zhai, Lingxiu Tong, Cuicui Wang, Zhiming Xie, Ke Zheng

**Affiliations:** State Key Laboratory of Reproductive Medicine and Offspring Health, Nanjing Medical University, Nanjing 211166, China

**Keywords:** lncRNA, function, screening, development, testis

## Abstract

Controversy continues over the functional prevalence of long non-coding RNAs (lncRNAs) despite their being widely investigated in all kinds of cells and organisms. In animals, lncRNAs have aroused general interest from exponentially increasing transcriptomic repertoires reporting their highly tissue-specific and developmentally dynamic expression, and more importantly, from growing experimental evidence supporting their functionality in facilitating organogenesis and individual fitness. In mammalian testes, while a great multitude of lncRNA species are identified, only a minority of them have been shown to be useful, and even fewer have been demonstrated as true requirements for male fertility using knockout models to date. This noticeable gap is attributed to the virtual existence of a large number of junk lncRNAs, the lack of an ideal germline culture system, difficulty in loss-of-function interrogation, and limited screening strategies. Facing these challenges, in this review, we discuss lncRNA functionality in organismal development and especially in mouse testis, with a focus on lncRNAs with functional screening.

## 1. Introduction

Mammalian cells house tens of thousands of long noncoding RNAs (lncRNAs) [1]. By far, dozens of them are well documented as being functional [2,3]. Needless to say, this only represents a minute fraction of the total. Relative to those shown in cell lines, a smaller number of lncRNAs are demonstrated to perform a function required for proper organismal development [4,5]. Even less recognized is the functionality of the mammalian testicular lncRNAs, few of which are fully validated as having a true role in either spermatogenesis or male fertility in knockout mouse models [6], although the testis expresses numerous species of lncRNAs [7,8]. Until now, how large a fraction of lncRNAs represent biological significance remains a central puzzle, fueling a long-lasting debate [9,10,11,12,13,14]. Some researchers argue that even though many useful lncRNAs do exist, the majority are not meaningful, regardless of whether or not they are transcribed excessively [15,16]. This opinion comes from several aspects. From a genomic perspective, recent global transcriptome profiling across eukaryotic organisms demonstrates that at least 85% of the genome is transcribed, which is far more than expected [17]. However, only 10% of the Pol II activities in yeast initiate from conventional promoters and the remaining events are noise [15]. Other evidence comes from several studies using mouse knockout models with the deletion of large DNA fragments that transcribe hundreds of transcripts. Nevertheless, these models reveal no apparent phenotype [18] and many functionless lncRNAs in animal models have been reported [19,20,21]. In contrast, other researchers claim that the functional discovery of lncRNA genes, which are difficult relative to protein-coding genes, is still in its infancy [22]. Still, a repository of functional lncRNAs has been compiled and appreciated [3]. It is thus conceivable that buried in the mass of junk transcripts could be a large proportion of functional lncRNAs that are, nevertheless, emerging like the tip of an iceberg [9,23].

Although much attention has been focused on the discovery of bona fide functional lncRNAs at the organismal level, this field is moving slowly. The human genome harbors a magnitude of lncRNA gene loci, ranging from 59,000 up to 102,000, based on different databases [24,25,26]. About 80% of the human genome is actively transcribed through the body’s development to adulthood [27]. While nearly 2000 lncRNAs have a putative functional association, most of them are described solely in cell lines and have yet to be studied in tissues [3]. To date, only a few lncRNAs have been functionally identified using animal models [2]. A thorough dissection of the functionality of a given lncRNA in organ development calls for effective and multipronged strategies. In this article, we review recent advances in screening and probing functional lncRNAs in organismal development, with an emphasis on testicular lncRNAs.

## 2. LncRNA Functionality: Prediction Rationale

With the advent of next-generation RNA sequencing technologies, the expressions of lncRNAs have been subjected to high-throughput analyses in various types of cells or tissues [4,28]. Compared with protein-coding genes, lncRNAs are expressed at lower levels while more specifically, i.e., in a cell type-, tissue-, developmental stage-, or disease state-specific manner [29,30,31]. Most lncRNAs are more tightly regulated than protein-coding genes, contending for a central role of lncRNAs in the cell state determination [32,33]. Thus, discerning functional subsets from a magnitude lncRNA pool is a priority [34] but has become a major hurdle in the field. Screening efforts based on common criteria such as physical gene locus proximity, lncRNA-gene co-expression, and sequence conservation, together with individualized paradigms, have pointed to the role of lncRNA in mammalian organ development [8]. For example, in *cis* co-expression of lncRNA and nearby genes has been widely applied to predicting functional lncRNAs. However, such a prediction strategy has been challenged in that co-activation of neighboring genes is driven by multiple lncRNA-associated mechanisms, such as enhancer-like DNA locus, splice sites, or transcriptional activity, but not exclusively by the lncRNA transcript itself [35]. Moreover, there are always contrasting variations across different species or organs, leading to generally limited efficacy in picking out bona fide functional lncRNAs [34]. Therefore, reconsidering traditional strategies to achieve a higher probability of mining out functional lncRNAs is now on the horizon.

## 3. Functional Screening: Cell Lines vs. Animal Models

Benefiting from fast and easy manipulation as well as low cost, cultured cell lines serve as an important platform to decrypt the functionality of individual lncRNAs. Moreover, these advantages make cell lines an ideal system for high-throughput lncRNA functional screening. Indeed, RNAi-, CRISPR/Cas9-, CRISPR/dCas9-, or CRISPR/Cas13-based screening strategies have successfully identified a dozen lncRNAs functioning in cell differentiation, growth, or response to stimuli [36,37,38,39]. While these seminal studies clearly demonstrate the role of lncRNAs, only a small subset of lncRNAs functioning in cell lines could be recapitulated in mouse models. A representative example is *Malat1*, which regulates growth-control genes at both the transcriptional and post-transcriptional levels [40]. Nevertheless, in vivo knockout mice show normal development and fertility [41]. Another example is *Evx1as*, which promotes EVX transcription in *cis* and regulates mesendodermal differentiation in pluripotent cell lines [42]. Nevertheless, *Evx1as*-ablated mice are viable with no obvious abnormality [19]. Similar contradictory outcomes from cell lines versus animal models are de facto often obtained and discussed in more cases [2].

Regardless of the long manufacturing period and heavy expenditure, the convincing level of lncRNA functional studies requires appropriate LOF manipulation on animals. Indeed, CRISPR/Cas9-based screening using different animal models, including C. elegans, Drosophila, zebrafish, and mouse, has been fruitful, but displays variable results since its emergence. Goudarzi et al. selectively tested 25 candidate lncRNAs based on their conservation, expression trait, and proximity to developmental regulators, showing that lncRNAs have no overt roles in zebrafish [20]. Schor et al. deleted 3 out of 362 lncRNAs with specific spatiotemporal expression in Drosophila embryogenesis but yielded no obvious phenotypes in normal conditions or under stress factors [43]. In addition, the genetic ablation of six cardiac-specific mouse lncRNAs with distinct transcriptional and epigenetic patterns resulted in no defects [44]. Despite these negative screening results in terms of the biological significance of tested lncRNAs, some other screens were more or less successful. Sauvageau et al. filtered out 18 lncRNAs with epigenetic modification for active transcription and conservation between mouse and human, of which three mutant strains displayed lethality and two exhibited growth defects [45]. A large-scale evaluation of 155 C. elegans knockouts of intergenic lncRNAs (lincRNAs) revealed phenotypes of 23 knockouts [46]. Another functional examination of 10 C. elegans knockouts revealed six lncRNAs required for normal development and fertility [47]. A canvass of lncRNA functional screens at the animal level is presented here in Table 1. As such, two groups of screens led to opinions that could be paradoxical regarding the prevalence of lncRNA functionality in animals, which has rather confused researchers. The in vivo lncRNA functional screens are far less fruitful than the progress in understanding their modes of mechanistic action. This gap is attributed to several explanations [2], including non-specific or off-target effects [48], transcript-independent mechanisms [35,49], functional redundancy [50], non-conserved function among species [51], and a stress- or disease-responsive role [4], as well as a missed phenotype [52,53].

Based on the research discussed above, cell lines and animal models have both advantages and disadvantages in functional screening. A combinational screen using both may allow for attaining a complementarity between large-scale screening ex vivo and the discovery of truly functional lncRNAs in vivo. Perhaps feasibly, for preliminary filtering, high-throughput RNAi or knockout approach can be exploited in primarily derived cell lines and, meanwhile, knockout models can be designed for endogenous validation of potential functional subsets. Indeed, several recent screens applied this dual-means strategy and therefore discovered dozens of lncRNAs in various cell lines and also in their corresponding organs [54,55,56] (Table 1).

**Table 1:** Strategy and efficiency of current knockout-based functional screening of lncRNAs in animals.

Organism	Queried Object of Organismal Development	Screening Criteria							Knockout Screening Tools	Knockout Screening Results	Reference
		Expression Specificity or Dynamicity	Expression Abundance	Conservation	Active Epigenetic Status	Physical Proximity with Regulators	Co-expression with Regulators	Other Screening Criteria			
C. elegans	Body development and fertility		√	√					CRISPR-Cas9	6/10	[47]
C. elegans	Body development and fertility								CRISPR-Cas9	23/155	[46]
Drosophila	Embryogenesis	√							CRISPR-Cas9	0/3	[43]
Drosophila	Spermatogenesis	√							CRISPR-Cas9	33/105	[57]
Zebrafish	Embryogenesis, viability, fertility	√		√		√			CRISPR-Cas9	0/25	[20]
Mouse	Intestine development	√						Pre-screen in cell lines	CRISPR-Cas9	1/1	[56]
Mouse	Neuroregeneration	√	√	√				Pre-screen in cell lines	CRISPR-Cas9	1/1	[54]
Mouse	Cardiomyocyte development	√	√					Pre-screen in cell lines	Homologous recombination	1/1	[55]
Mouse	Body development			√	√				Homologous recombination	5/18	[45]
Mouse	Heart development	√	√		√				CRISPR-Cas9	0/6	[44]
Mouse	Body development	√	√	√		√	√	Pre-screen in cell lines	CRISPR-Cas9	1/12	[19]

## 4. Testicular LncRNAs: Functionality

Over the past decade, an intense issue has raised the question of how functional lncRNAs originate from pervasive transcription [23,58]. A meaningful explanation is that functional lncRNAs occasionally evolve from a vast pool of non-functional transcripts via a mechanism similar to constructive neutral evolution [23]. In other words, only those organisms that produce a large quantity of junk RNAs possess many functional lncRNAs. Fitting this opinion, lncRNAs are highly expressed in nervous systems, where intensive research has revealed several subsets with physiological roles in neurogenesis, a field moving far beyond others [59].

Actually, the adult testis displays the highest transcriptome complexity among the tissues [8,60,61]. On one hand, during meiosis, chromatin relaxation activates a transcriptional burst of genic and intergenic RNAs, particularly lncRNAs, endo-siRNAs and pachytene piRNAs, along with transpositional shuffling to promote genomic and transcriptomic variability [62]. On the other hand, endo-siRNAs and pachytene piRNAs generated from antisense transcripts, usually from transposon sequences, guide the degradation of mRNAs and lncRNAs that could be useless or even deleterious, referred to as a molecular mechanism for genome-wide quality control [62,63]. Combined, the extensive transcription and targeted degradation of lncRNAs accelerate lncRNA evolution under robust screening through natural selection filters in mammalian spermatogenesis, giving rise to the thriving birth of young lncRNAs and preservation of optimized ones thereof [7,62]. All these clues highlight the significance of lncRNAs in spermatogenesis and hint at the existence of not a few functional ones. Although massive novel spermatogenic lncRNAs have been identified, most of them are biologically uncharacterized.

**Figure 1:**
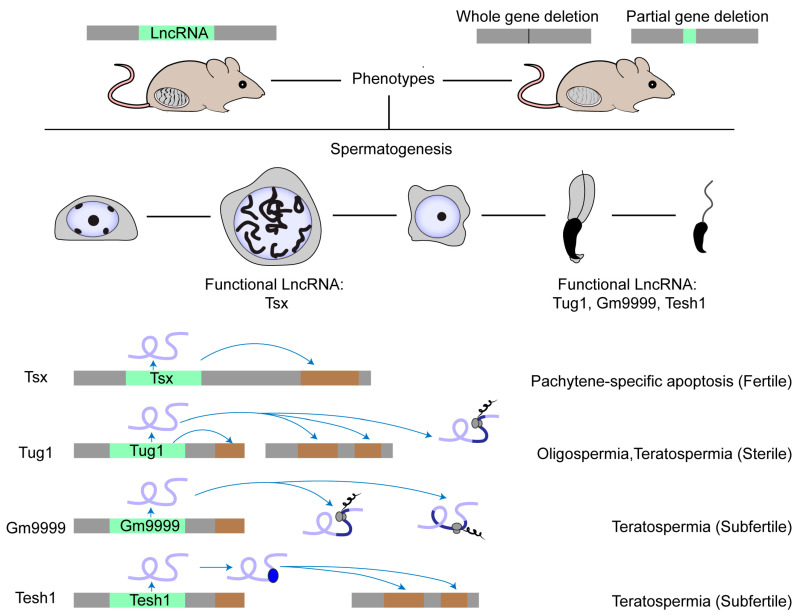
Physiologically functional lncRNAs in mouse testis. The physiological role of a few lncRNAs in mouse spermatogenesis has been studied by creating knockout mouse models. Most lncRNA knockouts delete the entire genomic locus. Other knockouts delete the functional element in the lncRNA locus, such as the ORF region in the *Gm9999* locus. So far, only four lncRNAs have been demonstrated as functional in mouse testis. *Tsx* is highly expressed in pachytene spermatocytes and *Tesh1* is mainly expressed in elongated spermatids. *Tug1*, *Gm9999*, and *Tesh1* knockouts exhibit teratospermia and impaired male fertility. Mechanistically, *Tsx* acts in *cis* and *Tesh1* in *trans*. *Tug1* could act in *cis*, in *trans*, or by an encoded protein. *Gm9999* executes its function through its two encoded polypeptides.

Several studies show the role of lncRNAs in male germ cells, which was summarized in recent reviews [6,64,65,66]. Here, we introduce functional lncRNAs with a focus on how to screen them in testis. So far, we still know little about lncRNA function in spermatogenesis, especially in mammals. There are only four lncRNA knockout models displaying obvious phenotypes (Figure 1). The first case is *Tsx* (testis-specific X-linked), whose knockout males were fertile with normal spermatogenesis [67]. Nonetheless, *Tsx* knockout resulted in a mild increase in the apoptosis of pachytene spermatocytes, an additional maternal-specific effect on litter size, and dysfunction of multiple other cell types. The second case is *Tesh1*, whose knockout males were subfertile with teratospermia and offspring with female-biased sex ratios [68]. The third case is *Tug1* (taurine-up-regulated gene 1), whose knockout males were sterile with decreased sperm counts and malformed sperm morphologies [69]. However, *Tug1* may not act merely as a lncRNA transcript because there are two additional attributive layers: as a *cis*-DNA regulator and as a protein-coding gene [69]. We still know little about whether these mechanisms alone or jointly contribute to its function in spermatogenesis [22]. The last case is *Gm9999*, which encodes two small peptides named Kastor and Polluks, respectively. Deletion of both peptides caused male subfertility with teratospermia and deletion of one peptide partially reproduced the phenotype, suggesting that *Gm9999* regulates spermatogenesis on the dependence of peptide generation [70]. These four rarely reported knockout mouse models that exhibit either mild or non-lncRNA transcript-exclusive effects on male fertility implicate a general difficulty in decoding the functional tacitness and complexity of testicular lncRNAs.

## 5. Testicular LncRNAs: Functional Screening

In one systematic study consisting of the functional screening of testicular lncRNAs, Wen et al. identified 128 testis-specific Drosophila lncRNAs and knocked out 105 of them [57]. Among these knockouts, 33 (31%) showed visual developmental defects in late male germ cells and a partial or complete loss of male fertility. This result supports the pervasive involvement of functional lncRNAs, at least in late spermatogenesis, and for the first time, supports a general relevance of lncRNAs to testis function. This study employed RNAi and rescue experiments to determine in *cis* or in *trans* regulation of gene expression and also to separate RNA-dependent lncRNA function from DNA-dependent effect. From an evolutionary viewpoint, a constant proportion (around 30% of the entire pool) of functional lncRNA sequences are likely accumulating all along, nearly equal in percentage to this study [57]. In another functional screen in mouse testis, Li et al. conducted global lncRNA expression profiling in six types of spermatogenic cells and subsequent filtering was based on protein-coding gene co-expression analysis and physical proximity with spermatogenic regulators [71]. They finally selected six lncRNA candidates and employed an shRNA-knockdown system in the testis to interrogate their in vivo function. As a result, two out of six candidate-depleted testes exhibited overt developmental defects in spermatogenesis. Fascinatingly, these screens in the testis of different species seem more efficient than in other tissues, supporting the aforementioned assumption that testicular transcriptome may comprise a relatively larger pool of functional lncRNAs.

## 6. Opportunities and Challenges

Due to our limited knowledge of lncRNA function in spermatogenesis, distinguished questions remain open. One notable question is whether lncRNAs are of significant importance in meiosis. The pervasively accessible chromatin observed during the meiotic progress includes intergenic regions [61], known as the major loci of lncRNA genes. As most stages of meiosis exhibit open chromatin states, tremendous lncRNAs are transcribed in spermatocytes, confirmed via single-cell RNA-seq analysis [72]. Moreover, several lncRNAs escape meiotic sex chromosome inactivation (MSCI) in pachytene spermatocytes and play potential roles in meiosis [73]. Although several mammalian spermatocyte-expressed lncRNAs have been functionally characterized, all of these studies rely on cell-based assays or have not produced an apparent phenotype in knockout mouse models [6,74,75,76]. More functional insights about meiotic lncRNAs are derived from yeast systems. One early study suggests a regulatory role of anti-sense lncRNA during the mitosis-to-meiosis transition [77]. Later studies identified a set of functional lncRNAs that regulate meiotic-specific chromosomal events at the transcriptional or post-transcriptional level [78]. Considering the potentially larger pool of lncRNAs and their complicated regulation in higher eukaryote systems, we reason that a considerable portion of lncRNAs perform a function in meiosis, which has yet to be explored.

Another obstacle that hampers our understanding of lncRNA functionality is our poor knowledge of the subcellular compartmentalization of lncRNAs in germ cells. It is well established that the localization of lncRNA is well suggestive of its cellular function [79,80]. Given its close relationship to molecular roles, subcellular localization would hopefully lift the cloud of mystery off lncRNA functionality. To date, lncRNAs have been detected in most of the general cellular organelles and granules [80]. However, our understanding of the subcellular localization of spermatogenic lncRNAs remains scarce. It is interesting to address how many lncRNAs are physically associated with germ-cell-specialized organelles and membraneless granules, such as the synaptonemal complex, the chromatoid body (CB), or acrosome. As mentioned previously, lncRNAs, along with RNA-binding proteins (RBPs) and other non-coding RNAs, constitute ribonucleoproteins (RNPs) and regulate synaptonemal complex formation directly [81]. Moreover, previous analysis of the CB component identified numerous lncRNA transcripts, suggesting their significance in CB assembly or function [82]. All of these findings hint that lncRNAs constitute spermatogenic RNPs and play a role in spermatogenesis. Furthermore, to what extent are lncRNAs functionally involved in the formation or function of these organelles in the process of piRNA biogenesis, meiosis, or sperm–egg recognition? A general method uses high-resolution imaging techniques, such as fluorescence in situ hybridization (FISH) and CRISPR/Cas13, to define lncRNA single-molecule localization [83,84,85]. An alternative method uses high-specificity biochemical methods, such as subcellular organelle purification coupled with RNA-seq or APEX-RIP, to detect high-throughput lncRNA-organelle associations [86,87]. Such methodological application to spermatogenic cells will speed our progress in decoding lncRNA function in the context of lncRNA-organelle association in spermatogenesis. In addition, technical advances in probing protein-bound lncRNAs hold equivalent promise at more refined layers of molecular interaction.

Most present screens focus on lincRNAs transcribed from intergenic regions, whereas other types of lncRNAs derived from introns (intronic lncRNAs) and gene regulatory regions (such as promoter lncRNAs and enhancer lncRNAs, elncRNAs/eRNAs) are rarely considered for screening, mainly because these lncRNAs are unstable and thus presumed to lack a function [88,89]. However, recent findings have challenged these stereotypes, showing that at least some of them play important biological roles [89,90]. Special attention should be paid to the new understanding of enhancers and enhancer-derived lncRNAs (elncRNAs). Enhancers control precise spatiotemporal gene expression and are thus the key regulators for cell specification and organ development [91,92]. Enhancers bind transcription factors that are presumed to interact with target promoters but more likely are responsible for the transcription of elncRNAs [93]. Enhancers are widely transcribed and many, if not most, lncRNAs are derived from enhancers [93,94,95], including some lncRNAs previously defined as a locus in intergenic regions [96]. elncRNAs can modulate DNA/histone modification as well as chromatin organization by binding to other RBPs or transcriptional factors [93,94,97]. Several elncRNAs are essential for organ development, such as *Evf2* [98], *Peril* [99], *Firre* [100], and *Maenli* [101], suggesting the physiological role of elncRNAs in vivo. Hence, elncRNAs are likely to be strong potential candidates in functional screening. Following elncRNA pre-screening, we must design thoughtful LOF strategies [101] and rigorously interpret phenotypes of animal models to distinguish the pure role of elncRNA as a complicated transcript influenced by its enhancer DNA locus or by its transcriptional activity [22].

In the testis, the expression of thousands of germline-specific genes gives rise to the most diverse and complex testicular transcriptome among all organs [61,102]. Discontinuous transcriptional states were coupled with unique chromatin remodeling across spermatogenesis [103]. To modulate these accurately monitored processes, germline-specific enhancers and associated regulation likely generally exist, despite the lncRNA expressed in testis are unlikely to be mainly derived from enhancers [71]. Indeed, the mitosis-to-meiosis transcriptome transition is driven by a switching from mitotic to meiotic super enhancers (SEs) [104]. Moreover, distinct regulation of meiotic Ses was observed on autosomes versus the sex chromosomes [104]. We believe that the testis harbors more functional elncRNAs than other tissues, which are only beginning to be unveiled with our understanding of spermatogenic enhancers.

## 7. Loss-of-Function Tools

Loss-of-function (LOF) methods are widely used to functionally interrogate or validate lncRNAs in cells and animals, including CRISPR-Cas gene-editing systems, RNA interference (RNAi), and antisense oligonucleotide (ASO). Several reviews compared their advantages and disadvantages and discussed their applicability based on different modes of action [22,34,105,106,107]. These LOF tools are being effectively utilized in the area of spermatogenesis. For example, CRISPR-Cas9-based knockout tools enabled a large-scale functional screen in Drosophila testis [57]. However, these tools cannot preclude that the DNA locus of a lncRNA plays a role, even if not dominantly, in producing a phenotype [108]. In the screening of mouse testicular lncRNAs, adeno-associated viral (AAV)-delivered shRNA-mediated knockdown was successfully applied in testis [71]. However, substantial knockdown efficiency of RNAi is not guaranteed in the nucleus [109,110], where lncRNAs are transcribed and may have the propensity to reside [88,111]. Superior to RNAi with respect to this point, ASO and CRISPR-Cas13 systems can be more efficient in targeting nuclear lncRNAs [110,112].

Furthermore, shRNA-based knockdown in testis showed the bias of targeting Sertoli cells rather than germ cells [113,114]. In addition, a virus-based delivery system needs intricate preparation of virus packaging, posing biohazard risks and artificial effects. Although siRNA avoids these shRNA-relevant caveats, its instability and transiency limit the use of siRNA for maintaining a long-term knockdown effect. In contrast, ASO can last several weeks and is widely used in disease treatment in mammals and humans [115,116]. Moreover, ASO can directly act on nascent transcripts to prevent transcription, whereas siRNA-mediated knockdown performs solely at the post-transcriptional level [117]. Thus, ASO can have a LOF effect on a lncRNA inclusive of its nascent or mature form. Our group recently established the technique of microinjecting ASO into living mouse testis to negatively modulate the levels of a target lncRNA [118]. Using FISH and a battery of other assays, we showed that ASO performed better than siRNAs in *Tsx* knockdown in recapitulating the phenotype of *Tsx* knockout. This study lays the groundwork for the fast interrogation of lncRNA functionality in testis. It is worth noting that ASO has a broad scope of applications not only in biological research but also in clinical trials [107], thanks to its merits, including easy nuclear accessibility, lack of need for delivery reagents, and low biological toxicity [119,120], which would be beneficial for reproductive health and therapy.

In general, RNAi serves as the most convenient approach for large-scale functional screening of lncRNAs both in vivo and in vitro [36,121]. Comparatively, ASO is more efficient, albeit more expensive, for nucleus-enriched lncRNA knockdown. Although the CRISPR-Cas9 gene editing completely ablates lncRNA transcripts and still represents the key benchmark to proclaim a functional lncRNA, we must carefully formulate the knockout designs and cautiously interpret the results when this lncRNA overlaps with other genes or regulatory elements.

## 8. Future Strategies for Screening

**Figure 2:**
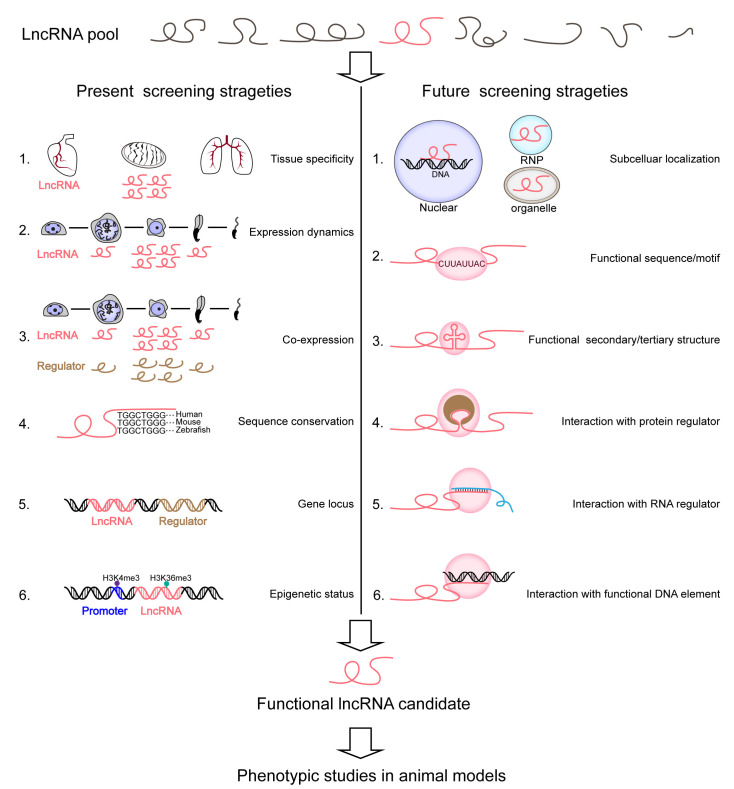
Schematic of present and future strategies to screen functional lncRNAs at the organismal level. We used mammalian testis as a model to depict these strategies. The present strategies tend to select lncRNA candidates based on tissue-specific, cell-type-predominant, or developmental dynamic expression patterns, co-expression with critical regulators, sequence conservation across species, genomic locus proximity to functional genes, and epigenetic marks for transcriptional activity (Table 1). Future strategies call for more attention to the molecular roles of lncRNAs including their subcellular localization, functional sequence, and structure, and interaction with other regulators or functional elements.

Overall, the present screening strategies are somewhat homogeneous and require more attempts to identify lncRNAs of biological meaning in development such as in spermatogenesis. It is widely accepted that surveyed lncRNAs can be predicted to be functional as a result of their multiple common features, which usually include expression abundance, sequence conservation, gene locus, and transcriptional states (Figure 2 and Table 1). The rank of lncRNA expression level, as one plausibly reasonable criterion, might always be overestimated in prioritizing putative functional candidates [17,122]. Another representative criterion worth discussion is the linkage of functionality and sequence conservation. However, even a conserved lncRNA may differ in the route of biogenesis or subcellular transport, leading to a distinct role in different species or organisms [51].

To select the best probable pool of candidate lncRNAs for functional interrogation using LOF approaches, we need to carefully gain as many properties as possible by tracing them from being transcribed at genomic loci to being post-transcriptional processed, and adopt rational and insightful strategies. Additional function-predictable features that might have been underestimated include, but are not limited to, subcellular localization, sequence/motif, secondary/tertiary structure, and interaction with protein regulator, RNA regulator, or DNA element (Figure 2). Although some of these topics are just beginning to be underpinned, inspired by some cutting-edge explorations [123,124,125,126], we hold promise to advance this field under joint efforts of scientific communities. For example, a recent study decoded that lncRNAs sharing similar small sequence (K-mers) contents have related functions although they lack a linear homology [127]. This work implies that short lncRNA elements are potential indicators for lncRNA functionality and can be used for high-throughput screening. For another delicate example, Khalil et al. profiled PRC2-associated lncRNAs, and the knockdown of certain subsets led to gene expression changes, with the up-regulated genes being globally silenced by PRC2 [128]. It is worth noting that PRC2-associated lncRNAs, such as HOTAIR and TUG1, were further demonstrated to function in vivo [69,129], suggesting that those lncRNAs that associate with central regulators of development can be investigated further. Moreover, other assays specialized in the detection of lncRNA-protein, lncRNA-DNA, or lncRNA-RNA interaction; secondary/tertiary structure; and subcellular localization will provide important clues for screening [130,131,132] (Figure 2).

In addition, many criteria for lncRNA characteristics are interconnected. The functional elements of lncRNA always determine its subcellular localization or protein chaperone [133,134]. LncRNA functional sequences/motifs are preconditioned to form secondary/tertiary structures in vivo, and both can be recognized by protein regulators to coordinate biological roles [5]. With our growing mechanistic understanding of lncRNAs, we will be able to more integratively and creatively screen functional lncRNAs, which are believed to greatly drive the field and ultimately unravel the functionality enigma of organismal lncRNAs.
